# Editorial: Smart Hydrogels in Tissue Engineering and Regenerative Medicine

**DOI:** 10.3389/fchem.2020.00245

**Published:** 2020-04-15

**Authors:** Xing Wang, Yanyu Yang, Yi Shi, Fei Jia

**Affiliations:** ^1^Beijing National Laboratory for Molecular Sciences, Institute of Chemistry, Chinese Academy of Sciences, Beijing, China; ^2^University of Chinese Academy of Sciences, Beijing, China; ^3^College of Materials Science and Engineering, Zhengzhou University, Zhengzhou, China; ^4^Sun Yat-sen University, Guangzhou, China; ^5^Massachusetts Institute of Technology, Cambridge, MA, United States

**Keywords:** hydrogel, tissue engineering, regeneration medicine, stimuli responsive, high performance

Tissue engineering, consisting of scaffolds, cells, favorable growth factors, and biomechanical stimulation, has been a most promising therapeutic strategy for tissue reconstruction and regenerative medicine. In general, an ideal tissue-engineered scaffold should be porous, non-toxic, with a matched biodegradable rate, high mechanics, and diffused nutrients and metabolite properties, which can effectively benefit the cell growth, proliferation, and migration, as well as the tissue vascularization process to promote tissue generation. Hydrogels, occurring abundantly with characteristics such as high-water absorption, biodegradation, adjustable porosity, and biocompatibility like that of the natural extracellular matrix (ECM), have been recognized as one of the most suitable scaffold biomaterials for tissue engineering and regenerative medicine. In addition, these scaffold-oriented hydrogels can also be utilized as idea carriers for embedding living cells, transporting tissue cells and growth factors, controlling degradation profiles and releasing stimulatory growth into the specific tissues at different time scales. Due to this, many researchers have been greatly interested in hydrogels with hierarchical structures to mimic the complex interaction of cells with their microenvironment at multiple length scales.

In this Research Topic, we have brought together 9 articles written by 60 authors containing 3 reviews and 6 original research articles. Review articles presented several up-to-date aspects of smart hydrogels in biomedical applications, such as the promoted tissue engineering (Liu et al.), improved transcatheter arterial chemoembolization (Chen Y.-P. et al.), and localized cancer therapy (Fan et al.) by means of their flexibly structural fabrications and functional organizations. As for the original research papers, many researchers were also focused on controllable hydrogel within a wide range of biomedical fields. For example, Bao et al. had prepared a self-assembled nanogel in solutions with a precise design of hierarchical structures using the feasible UV triggers for the potential drug carriers. Also, Hu et al. reported an injectable and compatible hyaluronic acid-based composite hydrogel with pH sensitive behaviors, which exhibited the low toxicity and acceptable mechanical property for liver cancer therapy and scaffolds for biomedical fields. In addition, Shi et al. reported a thermogelling system comprising poly(ω-pentadecalactone) (PPDL), poly(ethylene glycol) (PEG), and poly(propylene glycol) (PPG). The thermogels showed excellent thermal stability, fast response to temperature change, remarkable self-healing property and good biocompatibility, which showed high potential as a drug reservoir for a sustainable release profile of anti-tumor DOX payloads, and exhibited significant inhibition on the growth of tumor (Shi et al.). The other original research papers are covering important aspects of various hydrogels on tissue repair and regeneration, such as bone, hyaline cartilage, and osteoarthritis treatments. Zhang et al. demonstrated a controlled aspirin-sustained release system based on the uniform tetra-PEG hydrogels, which exerted favorable effects on the periodontal ligament stem cells-mediated bone regeneration, providing a new way of thinking about bone regenerative therapy. Wang et al. prepared a kartogenin-grafted PLGA-PEG-PLGA thermogel for achieving the cartilage regeneration and inhibiting the joint inflammation of arthritic knees in a rabbit model for osteoarthritis treatment. Chen Y.-R. et al. had synthesized a low-molecular-weight heparin-functionalized chitosan-chondroitin sulfate hydrogel, which could control the TGF-β3 release and promote the *in vitro* neocartilage formation for construction of tissue-engineered cartilage.

Therefore, the grand aim of this Research Topic is to underpin the importance of preparation, modification, and application of the various hydrogels for tissue engineering and regenerative medicine, which has been achieved by presenting a promising avenue in various fields and postulating real-world respective potentials. Collectively, owing to the intricate nature of emotions, studies aiming at its connection with the high performance of hydrogels are necessarily complex and multifocal. We sincerely hope that you will enjoy reading all the papers in this special edition.

## Author Contributions

All authors listed have made a substantial, direct and intellectual contribution to the work, and approved it for publication.

### Conflict of Interest

The authors declare that the research was conducted in the absence of any commercial or financial relationships that could be construed as a potential conflict of interest.

